# The upregulation of NLRP3 inflammasome in dorsal root ganglion by ten-eleven translocation methylcytosine dioxygenase 2 (TET2) contributed to diabetic neuropathic pain in mice

**DOI:** 10.1186/s12974-022-02669-7

**Published:** 2022-12-16

**Authors:** Wen Chen, Xiaotong Wang, Qingyu Sun, Yurui Zhang, Jing Liu, Tingting Hu, Weihua Wu, Chao Wei, Meng Liu, Yumeng Ding, Dianxin Liu, Yingzi Chong, Peipei Wang, Hongwei Zhu, Weihua Cui, Jiannan Zhang, Qian Li, Fei Yang

**Affiliations:** 1 Department of Neurobiology, School of Basic Medical Sciences, Capital Medical University, Beijing, 100069 China; 2 Department of Biochemistry and Molecular Biology, School of Basic Medical Sciences, Capital Medical University, Beijing, 100069 China; 3 Advanced Innovation Center for Human Brain Protection, Capital Medical University, Beijing, 100069 China; 4 Key Laboratory of Cancer Invasion and Metastasis Research, Capital Medical University, Beijing, 100069 China; 5 Department of Anesthesiology Beijing Tian Tan Hospital, Capital Medical University, Beijing, 100070 China; 6 Beijing Institute of Functional Neurosurgery, Xuanwu Hospital, Capital Medical University, Beijing, 100053 China; 7 International Acupuncture and Moxibustion Innovation Institute, Beijing University of Chinese Medicine, Beijing, 100029 China

**Keywords:** Diabetic neuropathic pain, TET2, NLRP3 inflammasome, Dorsal root ganglion

## Abstract

**Background:**

The nucleotide oligomerization domain (NOD)-like receptor family pyrin domain containing 3 (NLRP3) in dorsal root ganglion (DRG) contributes to pain hypersensitivity in multiple neuropathic pain models, but the function of the NLRP3 in diabetic neuropathic pain (DNP) and the regulation mechanism are still largely unknown. Epigenetic regulation plays a vital role in the controlling of gene expression. Ten-eleven translocation methylcytosine dioxygenase 2 (TET2) is a DNA demethylase that contributes to transcriptional activation. TET2 is also involved in high glucose (HG)-induced pathology.

**Methods:**

DNP was induced in mice via the intraperitoneal injection of streptozotocin (STZ) for five consecutive days and the mechanical threshold was evaluated in STZ-diabetic mice by using von Frey hairs. The expression level of the NLRP3 pathway and TET2 in DRG were determined through molecular biology experiments. The regulation of the NLRP3 pathway by TET2 was examined in in vitro and in vivo conditions.

**Results:**

In the present research, we first established the DNP model and found that NLRP3 pathway was activated in DRG. The treatment of NLRP3 inhibitor MCC950 alleviated the mechanical allodynia of DNP mice. Then we revealed that in STZ-diabetic mice DRG, the genomic DNA was demethylated, and the expression of DNA demethylase TET2 was increased evidently. Using RNA-sequencing analysis, we found that the expression of *Txnip*, a gene that encodes a thioredoxin-interacting protein (TXNIP) which mediates NLRP3 activation, was elevated in the DRG after STZ treatment. In addition, knocking down of *TET2* expression in DRG using *TET2*-siRNA suppressed the mRNA expression of *Txnip* and subsequently inhibited the expression/activation of NLRP3 inflammasome in vitro and in vivo as well as relieved the pain sensitivity of DNP animals.

**Conclusion:**

The results suggested that the upregulation of the TXNIP/NLRP3 pathway by TET2 in DRG was involved in the pain hypersensitivity of the DNP model.

**Supplementary Information:**

The online version contains supplementary material available at 10.1186/s12974-022-02669-7.

## Introduction

Diabetic neuropathic pain (DNP), one of the chronic complications of diabetes, is difficult to treat and results in a huge economic and psychological burden on patients [1, 2]. Although an increasing number of studies are focusing on DNP, its underlying mechanisms appear to be complex and not yet completely understood.

Inflammasomes are complexes comprising a sensor protein, an adaptor protein, and an effector protein pro-caspase-1 [3, 4]. Inflammasomes are categorized into two families—the nucleotide-binding domain, leucine-rich repeat-containing proteins (NLRs) [3, 4], and the absence in melanoma 2 (AIM2)-like receptors (ALRs) [5]. Once activated, its effector protein pro-caspase-1 produces cleaved-caspase-1, and subsequently cleaves pro-IL-1β to IL-1β, which then induces abnormal pain [6]. NLRP3 is a key molecule within the inflammasome, which can be activated by thioredoxin-interacting protein (TXNIP) and has attracted much attention in the field of pain [7–9]. Upregulation of NLRP3 inflammasomes of the sciatic nerve and dorsal root ganglion (DRG) is reportedly involved in DNP [10, 11]. However, the epigenetic regulatory mechanisms underlying the modulation of NLRP3 upregulation, remain to be elucidated.

Epigenetic regulation includes the modifications of DNA, histones, and non-coding RNAs. Recent research has confirmed that epigenetic regulation contributes to DNP [12–14]. DNA modification mainly includes DNA methylation and demethylation, both of which play vital roles in the regulation of gene expression. In mammals, DNA methylation is mainly mediated by the enzymes belonging to the DNA methyltransferase (DNMT) family, including DNMT1, DNMT3a, and DNMT3b, which transfer a methyl group to the C5 position of DNA cytosine to form 5-methylcytosine (5mC) [15]. In general, DNA methylation suppresses gene expression. Methylated DNA is demethylated by ten-eleven translocation methylcytosine dioxygenases (TETs), which are capable of oxidizing 5mC into 5-hydroxymethylcytosine (5hmC), 5-formylcytosine (5fC), and finally, 5-carboxycytosine (5caC), usually contributing to gene overexpression. TETs have three subtypes—TET1, TET2, and TET3 [16]. Our previous study indicated that the methylation level of the genomic DNA promoter region was altered in STZ-diabetic mice DRG [17]. Although it has already been validated that DNA methylation is involved in DNP, the focus of most research has been on DNMT-mediated DNA methylation [13], while the role of TETs in DNP had not been reported to date.

Using high levels of glucose to treat cultivate human mesangial cells (HMCs) is an often-used in vitro hyperglycemia cell model. Researchers observed a significant increase in levels of mRNA and protein of TET2, while the expressions of TET1 and TET3 exhibited no change [18]. Another study reported that using high levels of glucose to cultivate human umbilical vein endothelial cells (HUVECs), which were often used to study the effect of hyperglycemia on vascular endothelial in vitro, significantly increased the expression of NLRP3 [19]. These findings indicate that high glucose (HG) is capable of upregulating both TET2 and NLRP3. Moreover, it is reported that inflammasome genes undergo DNA demethylation during monocyte-to-macrophage differentiation and that *TET2*-siRNA inhibits this phenomenon [20]. These findings suggest that TETs-mediated DNA demethylation may influence the expression of inflammasome genes. In this context, the present study aimed to investigate whether TETs mediated the upregulation of NLRP3 inflammasome of DRG involved in DNP and its underlying mechanisms.

## Materials and methods

### Animals

Four hundred and fifty healthy wild-type male and 60 female C57BL/6 mice (age: 6–8 weeks) were procured from Charles River Laboratories. The animals were housed in cages (4–5 animals per cage) maintained at a temperature of 23 ± 2 °C and under 12-h light/12-h dark photoperiod and were fed with ordinary chow. Experiments were performed in accordance with followed ARRIVE and RIGOR guidelines [21]. All procedures were approved by the Experimental Animal Ethics Committee of Capital Medical University. Anesthesia and euthanasia of animals were consulted with the American Veterinary Medical Association (AVMA) Guidelines for the Euthanasia of Animals (2020).

### Type 1 diabetes mouse model

In order to establish the type 1 diabetes mouse models, the mice were subjected to overnight fasting, following which they were administered intraperitoneal (*i.p.*) injections of freshly prepared streptozotocin (STZ, 50 mg/kg/day, Sigma 0130) for five consecutive days. The control group mice were administered citrate buffer (5 mL/kg/day) for 5 days [22]. Fasting blood glucose levels were measured from the tail vein using the ACCU-CHEK^®^ Mobile blood glucose meter (Roche). If the fasting blood glucose level was above 11.1 mmol/L, the mouse was considered diabetic and used for subsequent analyses. The body weights of the mice were also recorded.

### Glucose tolerance test (GTT)

GTT was performed on non-fasted mice. Briefly, food was removed for 1 h, then mice were injected with 200 mg/mL of D-glucose (2 g/kg body weight, *i.p.*). Blood glucose levels were tested at 0, 15, 30, 45, 60, 90, 120 min post-glucose administration according to the previous report [23].

### HbA1c test

HbA1c was tested by a glycosylated hemoglobin assay kit (A056-1-1, Nanjing Jiancheng Bioengineering Institute) [24]. Briefly, blood of about 0.5–1 mL was harvested from the retro-orbital plexus in anticoagulant tubes, then centrifuged, and removed the supernatant. The sediment was washed in normal saline to obtain red blood cells solution. Double-distilled water was added to the above solution and mixed on a Vortex for 1 min to obtain dissolving blood. The absorbance of the dissolving blood was measured at 540 nm wavelength and the concentration of Hb was calculated according to the instruction of the kit. Acidified and hydrolyzed the dissolving blood and measured the absorbance at 443 nm. At last, the concentration of HbA1c was represented by the absorbance of every 10 g Hb (OD/10gHb). IFCC-HbA1c (%) = (OD/10gHb) × 0.001 + 0.0154; IFCC-HbA1c(mmol/mol) = 13 × IFCC-HbA1c (%) × 100–7.4.

### Intraepidermal nerve fiber density

Intraepidermal nerve fiber density (INFD) was assessed as described previously with minor modification [25]. Three randomly chosen 5-µm sections from each mouse were deparaffinized in xylene (1330-20-7, Shanghai Lingfeng Chemical Reagent, China), hydrated in decreasing concentrations of ethanol (100–75%), and rinsed in water. After antigen retrieval with citric acid (G1202, Servicebio, China) and endogenous peroxidase activity blocking with 3% H_2_O_2_ acid (Disinfection Technology, China), nonspecific binding was blocked by 10% goat serum (G1209, Servicebio) containing 3% BSA (G5001, Servicebio) for 30 min at room temperature. Then, PSD9.5 antibody (GB11277, ServiceBio, China) was added to the samples and incubated overnight at 4 °C. Slices were washed and incubated in secondary HRP-labeled antibodies (GB23303, ServiceBio) for 50 min at room temperature. Then, DAB chromogenic reaction was carried out with the commercial kit (G1211, Servicebio). Next, sections were counterstained with hematoxylin (G1340, Servicebio), dehydrated, and mounted in SweSuper clean Biomount medium (G1404, Servicebio). Intraepidermal nerve fiber profiles were counted blindly by independent investigators under a Nikon E100 microscope with a DS-U3 imaging system (Japan).

### Behavioral test

The mechanical threshold of hind paws was detected using calibrated von Frey hairs (Stoelting, Wood Dale, IL, USA) as described in our previous research works [17, 26, 27]. Briefly, the mice were placed on an elevated metal mesh floor and enclosed within a transparent plastic cage. The mice were allowed to habituate in this environment for a minimum of 30 min each day for 3 days prior to the test. Eight von Frey hairs (0.02, 0.04, 0.07, 0.16, 0.4, 0.6, 1.0, and 1.4 g) were selected. Each trial commenced with the 0.16 g von Frey hair and was delivered perpendicularly to the central plantar surface of either of the hind paws for 3–5 s. The 50% paw withdrawal threshold (PWT) was measured using the “up-and-down” method and the following formula [28]: 50% PWT(g) = 10^*X*^ ^+^ ^*kd*^/10^4^, where *X* denotes the value of the final von Frey hair used, *k* denotes the value from the pattern of positive/negative responses, and *d* is the average increment (in log units) among the von Frey hairs used.

Thermal sensitivity was measured using the Hargreaves method. Briefly, the mouse was allowed to adapt to the environment within a ventilated plexiglass cage with a glass floor. When the mouse was awake, and in a calm state, a radiant heat (15 W power) was applied to the center of either of the hind paws. The paw withdrawal latency (PWL) was recorded three times, and the average was used as the final threshold value. There was a minimum of 5 min interval between each set of consecutive measurements. A time threshold of 20 s was set to prevent possible tissue damage.

### Culture of mice DRG cells

DRG cells were isolated from naïve mice aged 4 weeks. Briefly, mice were anesthetized by isoflurane and then sacrificed quickly. The vertebral columns were removed and placed in an ice-cold DMEM solution (Gibco, C11995500BT). Then, about 150 DRGs were removed and pooled from 5 mice. DRGs were digested in 3% collagenase (Sigma, C9891) for 50 min and in 0.25% trypsin (Invitrogen, 15050065) for 10 min at 37 °C. The isolated DRG cells were seeded into poly-d-lysine (Sigma, P0899) coated 35-mm dishes and cultured in culturing medium [DMEM medium (Gibco, 11966025) supplemented with 10% FBS (Vistech, SE100-B), 5 mM glucose (Sigma, G7021), and 0.6 nM insulin (Wanbang Biopharma, 41-532K)] at 37 °C in a water-saturated atmosphere with 5% CO_2_ [29]. After 3 days of culturing, neurons were incubated in a different medium for an additional 24 h. Control group: the same with culturing medium. Mannitol (MT) group: DMEM medium supplemented with 10% FBS, 5 mM glucose, 0.6 nM insulin, and 20 mM MT (Energy Chemical, E100520). High glucose (HG) group: DMEM medium supplemented with 10% FBS and 25 mM glucose. The medium was changed every 12 h.

In another set of experiments, after isolating and culturing DRG neurons for three days in Neurobasal media (Gibco, 21103049) plus 2% B-27 supplement (Gibco, 17504044), DGR cells were cultured with the following solution, respectively, for 24 h: Neurobasal media (Gibco, 21103049) containing 25 mM glucose was used as the control group, and Neurobasal media with additional 20 mM MT were used as another control. In order to mimic hyperglycemia in vitro, Neurobasal with additional 20 mM glucose (total 45 mM) was used in the HG group. And the B-27 supplement was switched to the B-27 supplement minus insulin (Gibco, A1895601) in the HG group.

### RT-qPCR analysis

RT-qPCR assay was conducted as described before [30]. Total mRNA was extracted from cells or tissues using TRIzol reagent (15596026, life technologies, USA) from DRGs or primary DRG neuronal cultures. The cDNA was synthesized using a reverse transcription kit (R223-01, Vazyme, China), and qPCR experiments (7500, Applied Biosystem) were performed according to the instructions. After the reaction was completed, the sample was stored at 4 °C, and the resulting threshold cycle (CT) value was the number of cycles required when the fluorescence signal reached a set threshold. The relative expression amount of each pair of samples was calculated from (2^−ΔΔCT^).

**Table:** 

Gene	Forward	Reverse
*GAPDH*	TGTTCCTACCCCCAATGTGT	TGTGAGGGAGATGCTCAGTG
*TET2*	ATCCTTGCATTGGAGGGGTG	TTCCGGTCGGGATCGTTTAC
*TXNIP*	ACGTGTGTCAGTCTCTGCTC	AGTGTGTCGGGCCACAATAG
*NLRP3*	CCAGCCAGAGTGGAATGACA	ACAAATGGAGATGCGGGAGA
*Caspase-1*:	GGGACCCTCAAGTTTTGCC	GACGTGTACGAGTGGTTGTATT
*IL-1β*	AACTCAACTGTGAAATGCCACC	CATCAGGACAGCCCAGGTC

### Western blot analysis

Western blots were performed as previous reported [31]. Briefly, mice were euthanized with isoflurane anesthesia followed by rapid decapitation. The L3–L5 DRGs of the control and STZ-diabetic mice were retrieved (left and right sides, and a total of six DRGs from one mouse were pooled together) and subsequently disrupted using the RIPA lysis buffer. Briefly, the DRGs were sonicated and left on ice for 1 h, followed by centrifugation at 12,000*g* for 15 min at 4 °C to obtain the supernatant. An aliquot of 50 μg of protein was mixed with loading buffer and then placed in boiling water for 5 min. The proteins were separated on the SDS-PAGE gel and then transferred to a PVDF membrane. The PVDF membrane was blocked with 5% non-fat milk dissolved in TBST for 1 h at room temperature and subsequently incubated overnight with the following antibodies at 4 °C: rabbit anti-TET1 (1:1000, GeneTex), rabbit anti-TET2 (1:1000, Millipore), rabbit anti-TET3 (1:1000, ABclonal), rabbit anti-TXNIP (1:1000, Santa), rabbit anti-NLRP3 (1:1000), rabbit anti-caspase-1 (1:1000, ABclonal), rabbit anti-IL-1β (1:1000, ABclonal), and mouse anti-α-tubulin (1:10,000, Biodragon). Next, the membrane was washed 3 times with TBST (for 5 min each time) and then incubated with the HRP-labeled goat anti-rabbit or goat anti-mouse secondary antibodies. All antibodies had been diluted in 5% non-fat milk. Finally, the membrane was washed and exposed to ECL reagents for the detection of protein bands. The protein bands were scanned and analyzed using the Image J software.

### Immunofluorescence staining

As reported before [32], the mice were anesthetized with 1% pentobarbital sodium and then perfused with normal saline followed by a solution of 4% paraformaldehyde (PFA). The DRGs were post-fixed in 4% PFA for 4 h and then dehydrated sequentially in 20% and 30% sugar solutions. Each dehydrated DRG sample was excised into 10-µm-thick serial sections, which were then mounted on glass slides. After antigen retrieval and blocking with 5% fetal bovine serum, the DRG sections were co-incubated overnight at 4 °C with rabbit anti-TET2 antibody and DAPI with one of the following antibodies: mouse anti-NeuN (1:500, Sigma), mouse anti-NF200 (1:500, Sigma), or mouse anti-CGRP (1:500, Abcam). Subsequently, after washing the slides three times with phosphate buffered saline (PBS) (for 5 min each time), the sections were incubated with a mixture of Alexa Fluor 488 goat anti-rabbit IgG (H + L) and Alexa Fluor 568 goat anti-mouse IgG (H + L) (1:200, Invitrogen, Life technologies™, USA) 1 h in dark condition. Images were captured under a fluorescence microscope. We acquired at least 9 locations in the DRG per mouse: we quantified 3 images per section and 3 sections per mouse. We quantified at least 3 mice per experiment blindly.

### DNA dot-blot assay

The levels of 5-methylcytosine (5mC), 5-hydroxymethylcytosine (5hmC), 5-formylcytosine (5fC), and 5-carboxycytosine (5caC) were determined using DNA dot-blot assays [33]. Briefly, genomic DNA was extracted from the DRGs of mice according to the instructions of the kit (Solarbio, D1700). The extracted genomic DNA samples were categorized into three groups—62.5 ng, 125 ng, and 250 ng—based on their size and were then spotted on a nitrocellulose membrane using the Bio-dot microfiltration apparatus. The spotted membrane was placed in the ultraviolet crosslink equipment for 1 min and then at room temperature for 1 h. Subsequently, the membrane was blocked with 5% non-fat milk in TBST for 1 h, followed by incubation overnight at 4 °C with rabbit anti-5mC (1:1000, Merck), rabbit anti-5hmC (1:5000, Active Motif), rabbit anti-5fC (1:5000, Active Motif), and rabbit anti-5caC (1:5000, Active Motif) in TBST buffer with 5% non-fat milk. After washing the membrane three times with TBST (for 5 min each time), it was incubated with goat anti-rabbit secondary antibody (1:1000, HRP labeled) dissolved in 5% non-fat milk at room temperature for 1 h. The membrane was washed again three times (for 5 min each time) and exposed to the ECL reagent for detection. The intensity of the dot-blot image was analyzed using the Image J software. Meanwhile, the control DNA dot-blot membrane spotted as the same before was placed in 0.02% methylene blue diluted in 0.3 M sodium acetate to stain the DNA.

### Intrathecal injection, intraperitoneal injection, and in vitro siRNA transfection

The mice (10–12 weeks) were anesthetized using isoflurane and then placed in a prone position. A lumbar puncture was performed using a 30-gauge needle connected to a 25-µL Hamilton microsyringe as reported before [31]. The NLRP3 inhibitor MCC950 (InvivoGen, inh-mcc) and the siRNA (Santa Cruz, TET2-siRNA: sc-154205; Scr-siRNA: sc-44238) were administered at an injection volume of 5 µL for each. During the process, if the mouse demonstrated tail movement, the intrathecal injection was successful.

For intraperitoneal injection, MCC950 (1 mg/kg, 3 mg/kg, 10 mg/kg) or CY-09 (5 mg/kg, Sigma, SML2465) were injected by 1 mL sterile insulin syringe at 4 weeks after STZ injection.

To silence *TET2* in DRG neurons, after three days of culture, cells were incubated with Opti-MEM™I (Gibco, 31985-070), Lipofectamine™ RNAiMAX (Invitrogen, 13778-150), and TET2-siRNA or Scr-siRNA for 12 h. Then the medium was changed to HG culturing medium for an additional 24 h.

### RNA-sequence analysis

The total RNA was extracted from the DRG sample using the TRIzol reagent [34]. Agilent 2100 Bioanalyzer was used for determining the concentration of the extracted RNA. NanoDrop was employed to detect the purity of the extracted RNA. Sequencing libraries were generated using NEBNext^®^ Ultra™ RNA Library Prep Kit for Illumina^®^ (NEB, USA) by following the manufacturer’s recommendations. Index codes were added to attribute the sequences to the corresponding samples. Subsequently, the prepared libraries were sequenced on the Illumina Hiseq 2000/2500 platform (IGENECODE, Beijing). The expression level of each transcript was calculated through FPKM (fragments per kilobase of the transcript sequence per million base pairs sequenced). Hierarchical clustering was performed to analyze the differentially expressed genes (DEGs). When analyzing the RNA-seq data, we set the fold change above 1.5 with a probability above 0.75 and considered the level of mRNA changes significant. In the Kyoto Encyclopedia of Genes and Genomes (KEGG) analysis, when the *p* value was lower than 0.05, the signaling pathway was considered significantly enriched.

### Statistical analyses

The data were expressed as mean ± SD and analyzed in GraphPad Prism software. Two-tailed Student’s *t* test followed by Tukey's multiple comparisons test was used to compare two groups. In order to compare multiple groups, one-way analysis of variance (ANOVA) followed by Tukey’s post hoc test or two-way ANOVA followed by Bonferroni’s post hoc test was performed. A *p-*value of < 0.05 was considered statistically significant. All experiments were repeated at least three independent times except the RNA-seq experiment.

## Results

### Intraperitoneal injection of STZ for inducing DNP in mice

**Fig. 1:**
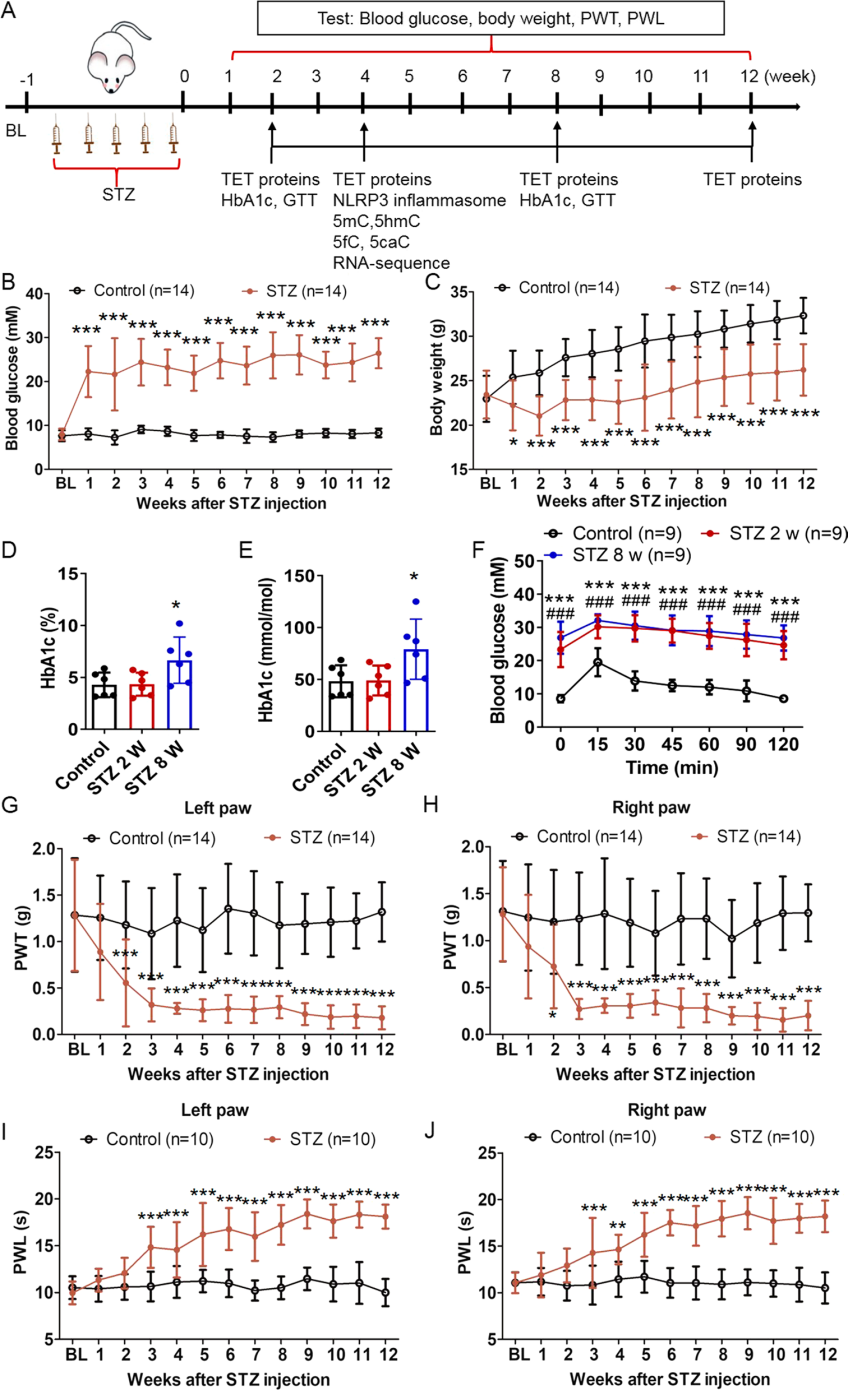
Intraperitoneal injection of STZ induced DNP. **A** Flowchart of the experiment design. **B** Fasting blood glucose levels were measured in the STZ-treated and control mice. *n* = 14, ***p < 0.001, two-way ANOVA with Bonferroni post hoc test. **C** Bodyweight in the STZ-treated and control mice. *n* = 14, *p < 0.05, ***p < 0.001, two-way ANOVA with Bonferroni post hoc test. **D**, **E** The level of HbA1c in STZ-treated and control mice. *n* = 6, * *p* < 0.05, one-way ANOVA with Tukey’s post hoc test. **F** The glucose tolerance test in STZ-treated and control mice. *n* = 9, *** *p* < 0.001 STZ 8 w versus control; ###*p* < 0.001 STZ 2 w versus control, two-way ANOVA with Bonferroni post hoc test. **G**, **H** Mechanical allodynia in the left and right paws of the STZ-treated mice. *n* = 14, *p < 0.05, ***p < 0.001, two-way ANOVA with Bonferroni post hoc test. **I**, **J** Thermal hyposensitivity in the left and right paws of the STZ-treated mice. *n* = 10, **p < 0.01, ***p < 0.001, two-way ANOVA with Bonferroni post hoc test. Experiments were repeated at least 3 times independently

In order to establish the diabetic mouse models, STZ injections were administered to wild-type C57BL/6 male mice (6–8 weeks old) for 5 days (Fig. 1A). As expected, the fasting blood glucose levels were significantly increased in the STZ-treated mice (Fig. 1B). Moreover, the bodyweight of the STZ-treated mice was lower than that of the control mice (Fig. 1C). Compared to the control mice, the HbA1c level of STZ-treated mice did not change at 2 weeks, but significantly increased after 8 weeks post-STZ injection (Fig.1D and E). The glucose tolerance of STZ-treated mice was also significantly impaired after 2 and 8 weeks post-STZ injection (Fig. 1F).

The von Frey hairs were used for determining the presence of mechanical allodynia in STZ-diabetic mice. It was observed that at 2 weeks post-STZ injection, the threshold of response to mechanical stimulation in the left and right paws was dramatically lower than that in the control mice (Fig. 1G and H). In addition, the thermal sensitivity of the STZ-diabetic mice was analyzed, and it was observed that these mice exhibited thermal hyposensitivity (Fig. 1I and J). According to these results, the diabetic mice exhibited significant hypersensitivity to mechanical stimulation and, therefore, the diabetic neuropathic pain mouse models were successfully established. Since the pain sensitivity in the left and right paws was almost the same, in the subsequent analyses, the average value was used as the final data. We next assessed the intraepidermal nerve fiber density (IENFD) in control mice and diabetic mice. The IENFD in diabetic mice was notably reduced by around 50% compared to control mice at 8 weeks post-STZ injection (Additional file 1: Fig. S1A and B). The significant sensory (PGP-positive epidermal fibers) denervation in footpads correlates with reduced sensitivity to mechanical and heat stimuli at the footpads suggesting that diabetic mice show neuropathy at 8 weeks post-STZ injection.

### Upregulated NLRP3 inflammasome involved in DNP

**Fig. 2:**
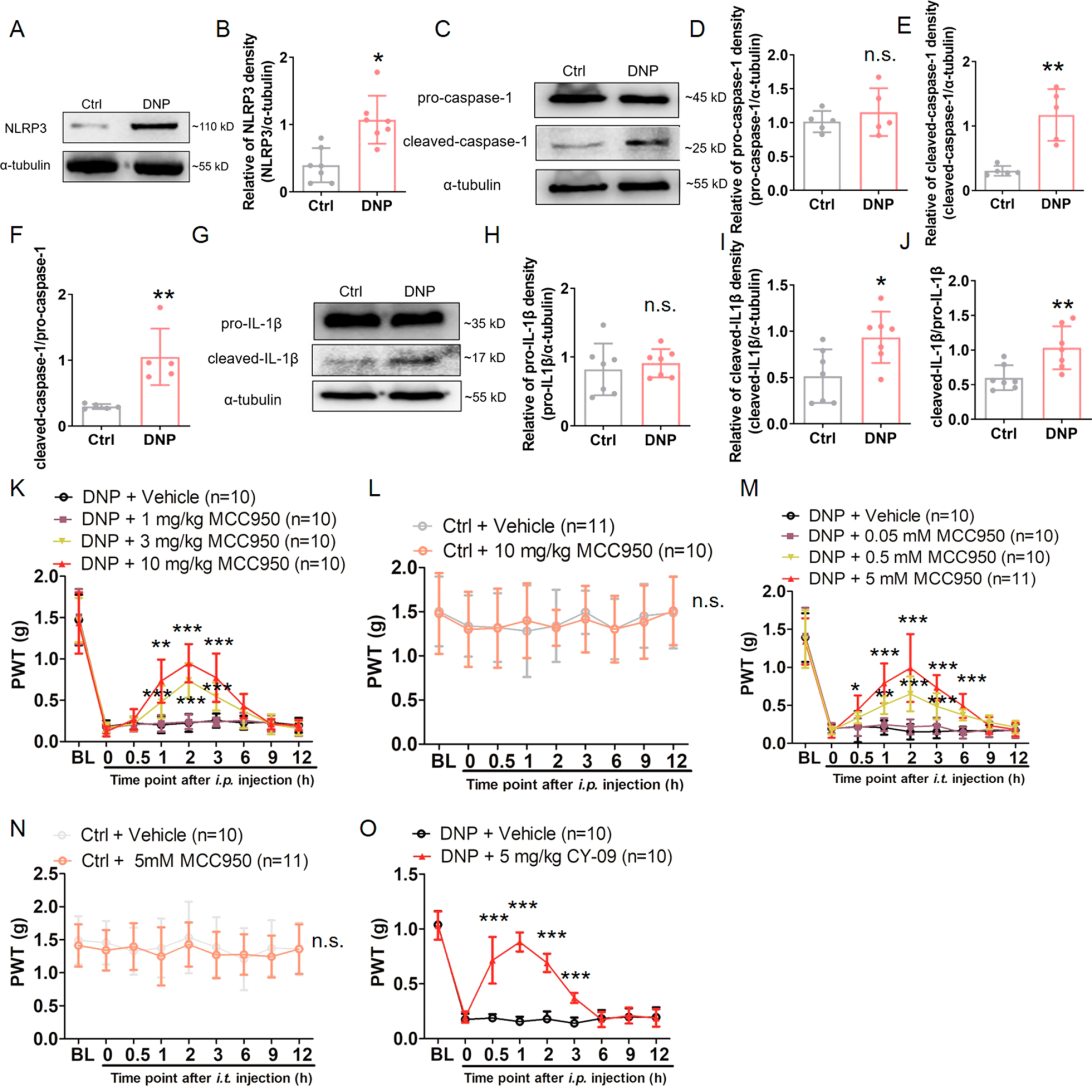
NLRP3 inflammasome was increased in STZ-diabetic mice DRG and was involved in pain sensitivity. **A** Representative Western blot bands for NLRP3 and α-tubulin at four weeks post-STZ injection in control and DNP group. **B** Statistical analysis for NLRP3 and α-tubulin. *n* = 7, *p < 0.05, unpaired *t*-test. **C** Representative Western blot bands for pro-caspase-1, cleaved-caspase-1, and α-tubulin at four weeks in control and DNP group. **D** Statistical analysis for pro-caspase-1 and α-tubulin. n.s., not significant. **E** Statistical analysis for cleaved-caspase-1 and α-tubulin. *n* = 5, **p < 0.01, unpaired *t-*test. **F** Statistical analysis for cleaved-caspase-1 and pro-caspase-1. *n* = 5, **p < 0.01, unpaired *t-*test. **G** Representative Western blot bands for pro-IL-1β, cleaved-IL-1β, and α-tubulin at 4 weeks post-STZ injection in control and DNP group. **H** Statistical analysis for pro-IL-1β and α-tubulin. n.s., not significant. **I** Statistical analysis for cleaved-IL-1β and α-tubulin. *n* = 7, *p < 0.05, unpaired *t-*test. **J** Statistical analysis for cleaved-IL-1β and pro-IL-1β. *n* = 7, **p < 0.01, unpaired *t-*test. **K** Intraperitoneal injections of 3 mg/kg and 10 mg/kg MCC950 significantly relieved pain sensitivity in the STZ-diabetic mice, while the same did not happen with 1 mg/kg MCC950 injection. *n* = 10, **p < 0.01, ***p < 0.001 versus the vehicle group, two-way ANOVA with Bonferroni post hoc test. **L** Intraperitoneal injection of 10 mg/kg MCC950 did not affect the behavior of control mice, *n* = 10–11, two-way ANOVA with Bonferroni post hoc test. n.s., not significant. **M** Intrathecal injections of 0.5 mM and 5 mM MCC950 significantly relieved pain sensitivity in the STZ-diabetic mice, while the same did not happen for 0.05 mM MCC950 injection. *n* = 10–11, *p < 0.05, **p < 0.01, ***p < 0.001 versus the vehicle group, two-way ANOVA with Bonferroni post hoc test. **N** Intrathecal injection of 5 mM MCC950 did not affected the behavior of control mice. *n* = 10 –11, two-way ANOVA with Bonferroni post hoc test. n.s., not significant. **O** Intraperitoneal injection of 5 mg/kg of CY-09 significantly relieved pain sensitivity in the STZ-diabetic mice. *n* = 10, ***p < 0.001 versus the vehicle group, two-way ANOVA with Bonferroni post hoc test. Experiments were repeated at least 3 times independently

While the behavior of the STZ-diabetic mice was stable after 3 weeks after STZ injection as shown in Fig. 1, the role of NLRP3 inflammasome in DNP was investigated by performing Western blot analysis to evaluate the expressions of NLRP3, pro-caspase-1, cleaved-caspase-1, pro-IL-1β, and cleaved-IL-1β, at four weeks after STZ injection. As expected, the expression of NLRP3 increased significantly in the DRG of the DNP group (Fig. 2A and B). When the NLRP3 inflammasome was activated, pro-caspase-1 was activated and produced cleaved-caspase-1. The Western blot analysis also revealed that there was no significant change in the expression of pro-caspase-1, while the expression of cleaved-caspase-1 was significantly upregulated compared to control mice (Fig. 2C–F). Moreover, the ratio of cleaved-caspase-1/pro-caspase-1 in the DRG of the DNP group also increased dramatically compared to the control mice (Fig. 2C–F). Cleaved-caspase-1 is capable of inducing the conversion of pro-IL-1β into cleaved-IL-1β. Furthermore, the Western blot analysis revealed that there was no significant change in the pro-IL-1β expression, while the expression of cleaved-IL-1β had evidently increased, compared to control mice at four weeks post-STZ injection (Fig. 2G–J). When the ratio of cleaved-IL-1β/pro-IL-1β was compared between DNP and control mice DRG, it was observed that the ratio was significantly increased in the DNP group (Fig. 2G–J). These results indicated the activated status of the NLRP3 inflammasome in the DRG at 4 weeks post-STZ injection when the DNP was stable.

In order to explore the potential contribution of the upregulation of the NLRP3 inflammasome within the DRG in the mechanical allodynia occurring in STZ-diabetic mice, the effects of MCC950, a selected small-molecule NLRP3 inflammasomes inhibitor [7, 8], were investigated by injecting MCC950 intraperitoneally into both control and DNP group at four weeks after models were constructed. PWT behavior tests were conducted at 0.5, 1, 2, 3, 6, 9, and 12 h after the injection. As depicted in Fig. 2K, the intraperitoneal injection of 10 mg/kg MCC950 to the DNP group presented a significant relief in the pain response to mechanical stimulation, with a peak at 2 h, compared to the DNP + vehicle group. The intraperitoneal injection of 3 mg/kg MCC950 to the DNP group also relieved the pain response in comparison to the DNP + vehicle group (Fig. 2K). On the contrary, the intraperitoneal injection of 1 mg/kg MCC950 presented no effect on the mechanical allodynia in the DNP group compared to the DNP + vehicle group (Fig. 2K). These results indicated that the analgesic effects of MCC950 were dependent on its dose. In control mice, the intraperitoneal injection of 10 mg/kg MCC950 exerted no effect on the PWT behavior compared to the control + vehicle group (Fig. 2L), indicating that MCC950 did not affect normal pain sensitivity.

Intraperitoneal injection affects the whole body, while intrathecal injection preferentially affects DRG and the spinal cord. Therefore, in order to further investigate the role of NLRP3 inflammasome in the DNP behavior of mice in DRG, we intrathecally injected 0.05 mM, 0.5 mM, and 5 mM of MCC950, and then subjected them to PWT behavior tests at 0.5, 1, 2, 3, 6, 9, and 12 h after the injections. As depicted in Fig. 2M, the intrathecal injection of 5 mM MCC950 significantly alleviated the pain response, with a peak at 2 h after the injection, compared to the DNP + vehicle group. The intrathecal injection of 0.5 mM MCC950 also relieved the pain behavior in the DNP group compared to the vehicle group (Fig. 2M). On the contrary, the intrathecal injection of 0.05 mM MCC950 presented no effect on mechanical allodynia in the DNP group compared to the vehicle group (Fig. 2M). These results reinforced that the analgesic effects of MCC950 were dependent on its dose. In control mice, the intrathecal injection of 5 mM MCC950 presented no effect on the PWT behavior compared to the vehicle group (Fig. 2N), which reinforced that MCC950 did not alter the normal pain sensitivity.

Furthermore, to confirm that MCC950-resolved pain was due to the inflammasome inactivation and avoid the off-target effect of MCC950, we injected CY-09, another NLRP3 inflammasome inhibitor, into STZ-diabetic mice and examined the pain behavior. Consistent with the results gained from injecting MCC950, CY-09 also relieved the pain behavior effectively (Fig. 2O).

Overall, the above results suggested that upregulation of the NLRP3 inflammasome of DRG contributed to DNP at early time points.

### Whole genomic DNA was demethylated in STZ-diabetic mice DRG

**Fig. 3:**
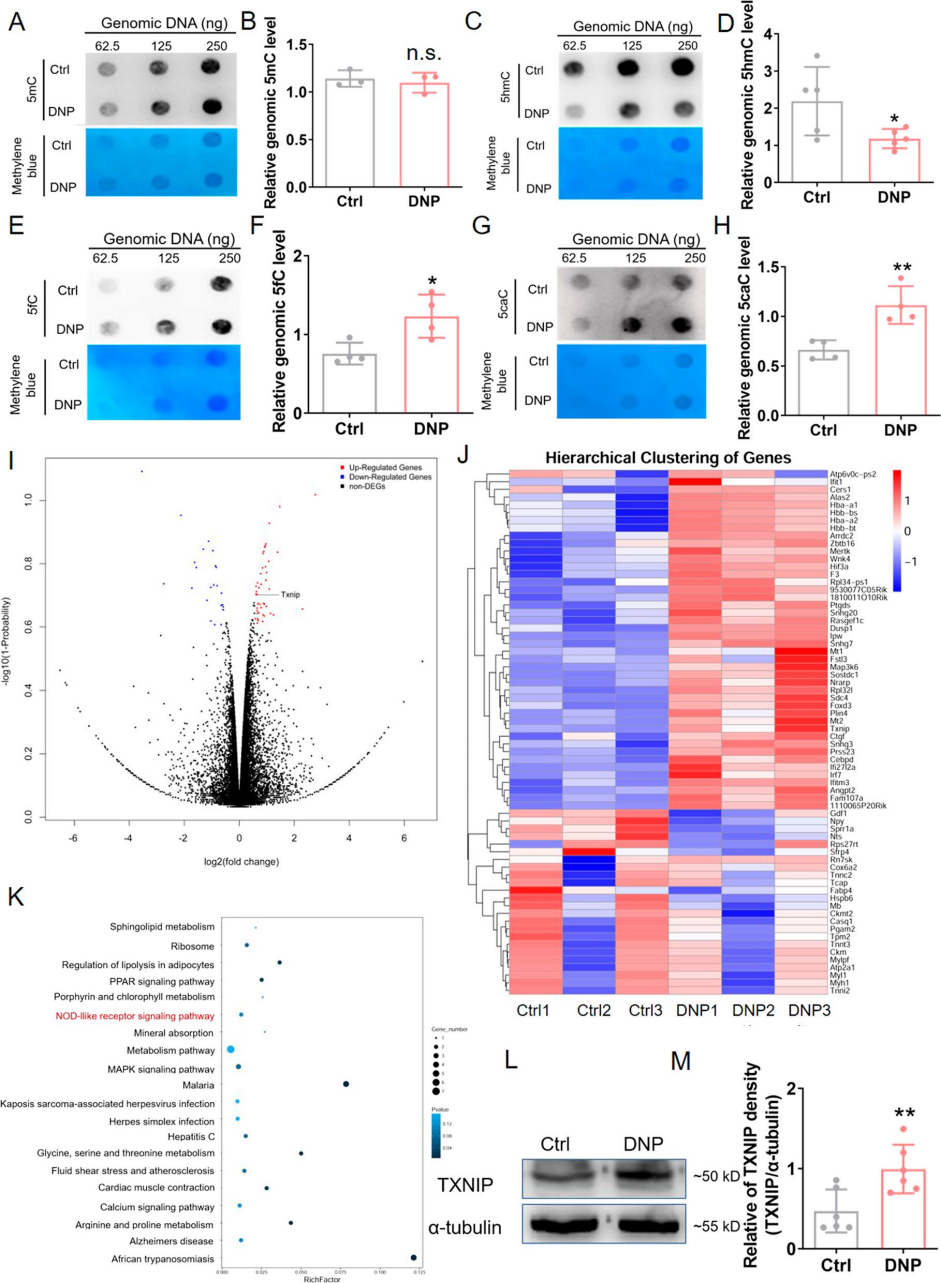
Genomic DNA in the STZ-diabetic mice DRG was demethylated. **A** Genomic DNA 5mC was examined in both control and DNP group. Methylene blue staining was performed to maintain the quality of the DNA samples when transferred to membrane blots. **B** Statistical analysis for 5mC. *n* = 3, unpaired *t*-test. n.s., not significant. **C** Genomic DNA 5hmC was examined in control and DNP group, and methylene blue staining was performed to maintain the quality of the DNA samples when transferred to membrane blots. **D** Statistical analysis for 5hmC. *p < 0.05, *n* = 5, unpaired *t-*test. **E** Genomic DNA 5fC was examined in control and DNP group, and methylene blue staining was performed to maintain the quality of the DNA samples when transferred to membrane blots. **F** Statistical analysis for 5fC. *p < 0.05, *n* = 4, unpaired *t-*test. **G** Genomic DNA 5caC was examined in control and DNP group, and methylene blue staining was performed to maintain the quality of the DNA samples when transferred to membrane blots. **H** Statistical analysis for 5caC. **p < 0.01, *n* = 4, unpaired *t-*test. **I** Volcano plot of DNP versus control mice DRG. *n* = 3. **J** Heatmap analysis of the differently expressed mRNAs in DNP versus control mice DRG. *n* = 3. **K** KEGG pathway enrichment analysis revealed the top 20 signaling pathways. **L** Representative Western blot bands for TXNIP and α-tubulin at four weeks in control and DNP group. **M** Statistical analysis for TXNIP and α-tubulin. *n* = 6, **p < 0.01, unpaired *t*-test. Experiments were repeated at least 3 times independently except the RNA-sequencing experiment

The NLRP3 inflammasome upregulation/activation is detected in the DRG of DNP animals, and DNA demethylation plays an important role in activating the gene expression. So, we assessed the methylation of the whole genomic DNA in the DRG. The DNA dot-blot assays revealed that the whole genomic DNA was demethylated in the DRG at four weeks post-STZ injection. As depicted in Fig. 3A–H, the expression of 5-methylcytosine (5mC) in the DNP group did not exhibit a significant change compared to that in the control mice. On the other hand, the expression of 5-hydroxymethylcytosine (5hmC) in the DRG genomic DNA was significantly decreased in the DNP group compared to that in the control group. The expressions of 5-formylcytosine (5fC) and 5-carboxycytosine (5caC) were significantly increased in the DNP group compared to those in the control group.

In general, DNA demethylation contributes to gene transcription. Therefore, the expression profile of the whole genome mRNA was analyzed using RNA-sequencing. The obtained data revealed a total of 68 genes that differed between the DNP and the control mice at 4 weeks after STZ injection (at least a 1.5-fold change with a probability above 0.75). Among these genes, 45 genes were significantly upregulated, and 23 genes were dramatically downregulated (Fig. 3I and J). In order to better understand the differences in a single sample, hierarchical clustering of differentially expressed genes was performed, and the results are presented in Fig. 3J. Moreover, the Kyoto Encyclopedia of Genes and Genomes (KEGG) analysis was performed, and when the *p* value was lower than 0.05, and the signaling pathway was considered significantly enriched. In Fig. 3K, the top 20 enriched pathways were shown, and we noticed that the nucleotide oligomerization domain (NOD)-like receptor signaling pathway was in the list of altered pathways. The NOD-like receptor signaling pathway included a gene named *Txnip*, and its protein product thioredoxin-interacting protein (TXNIP) is an endogenous activator of the NLRP3 inflammasome. Using Western blot analysis, it was revealed that the expression of the TXNIP protein was increased in the DNP group compared to the control group (Fig. 3L and M).

The above results suggested that DNA demethylation might play a vital role in mediating the involvement of NLRP3 inflammasome through regulating *Txnip* gene expression in DNP.

### TET2 was significantly increased in STZ-diabetic mice DRG

**Fig. 4:**
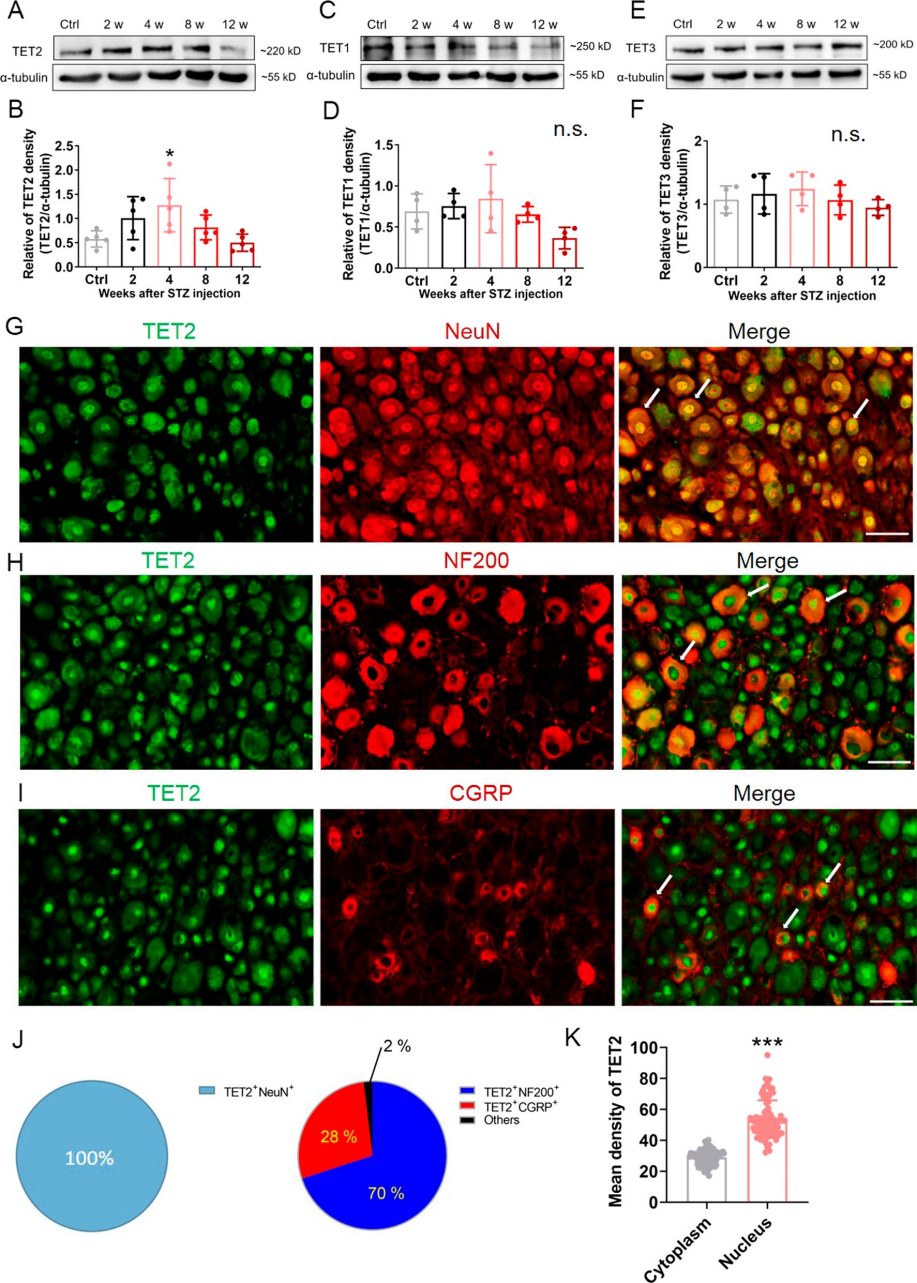
TET2 was upregulated in the DRG neurons of STZ-diabetic mice. **A** Representative Western blot bands for TET2 and α-tubulin at different time points in control and DNP group. **B** Statistical analysis of the relative band density of TET2. *n* = 5, *p < 0.05, one-way ANOVA with Tukey’s post hoc test. **C** Representative Western blot bands for TET1 and α-tubulin at different time points in control and DNP group. **D** Statistical analysis of the relative band density of TET1. *n* = 4, n.s., not significant, one-way ANOVA with Tukey’s post hoc test. **E** Representative Western blot bands for TET3 and α-tubulin at different time points in control and DNP group. **F** Statistical analysis of the relative band density of TET3. *n* = 4, n.s., not significant, one-way ANOVA with Tukey’s post hoc test. **G** Double staining of TET2 and NeuN. **H** Double staining of TET2 and NF200. **I** Double staining of TET2 and CGRP. Scale bar = 50 µm. Arrows indicate the representatives of double-stained cells. **J** The ratio of NeuN^+^, NF200^+^, CGRP^+^ cells in TET2^+^ cells. *n* = 3. **K** The mean density of TET2 in nucleus was higher compared to cytoplasm. *n* = 90 cells, *n* = 3 mice, ***p < 0.001, unpaired *t*-test. Experiments were repeated at least 3 times independently

In the mammalian genome, DNA demethylation is mediated mainly by TETs. The Western blot analysis results revealed that the expression of TET2 in STZ-diabetic mice was significantly increased at 4 weeks after STZ injection compared to that in the control mice (Fig. 4A and B), while the expressions of TET1 and TET3 exhibited no statistical change compared to the control group (Fig. 4C–F). These results indicated that TET2, and not TET1 and TET3, within the DRG, might be sensitive to high glucose (HG), and play an important role in the DNA demethylation in STZ-diabetic mice.

Since only TET2 was upregulated, but not the other TETs, we next assessed the cell source of TET2 in mice DRG. The immunofluorescence staining results revealed that in naïve mice DRG, TET2 was highly co-expressed with NeuN, which is a marker of neuron nucleus (Fig. 4G). Moreover, TET2 was also co-expressed with NF200 (a myelinated large-diameter neuronal marker) and CGRP (a peptidergic small-diameter neuronal marker) (Fig. 4H and I). We further quantified the ratio of TET2^+^ and NeuN^+^, NF200^+^, or CGRP^+^ cells, and the fluorescence intensity of TET2 in the nuclear and cytoplasm fraction. We found that almost all TET2^+^ cells were also NeuN^+^, and the majority of TET2^+^ cells were NF200^+^ neurons (Fig. 4J). Additionally, TET2 was accumulated in the nuclear portion than that in the cytosol portion (Fig. 4K). These results indicated that TET2 was mainly located in the nucleus of the DRG neurons and the nuclear localization of TET2 was consistent with its DNA demethylase function.

### TET2 regulated NLRP3 activation through upregulation of *Txnip*

**Fig. 5:**
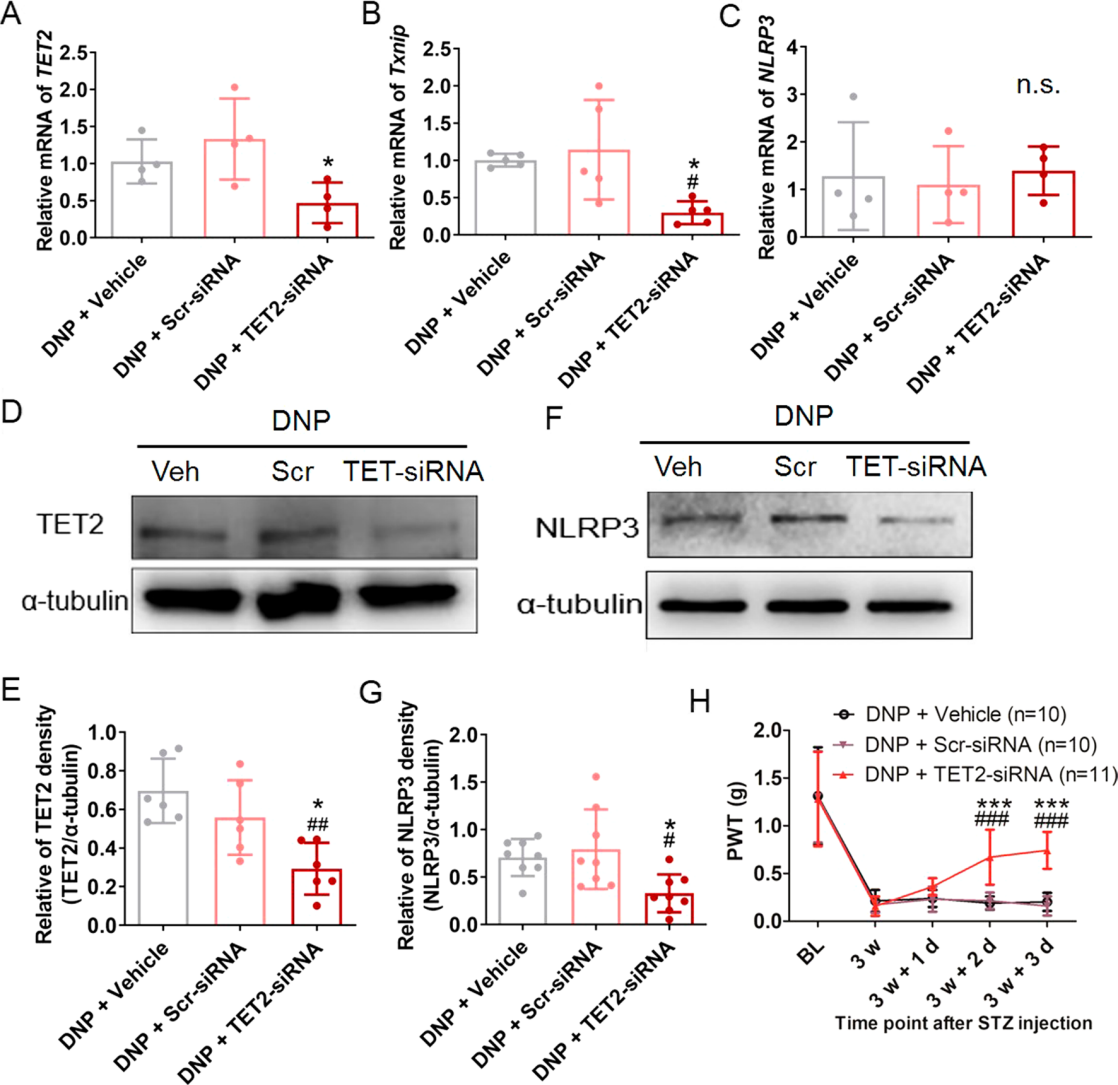
TET2 regulated the TXNIP/NLRP3 expression in STZ-diabetic mice DRG. **A**–**C** Intrathecal injection of *TET2*-siRNA and the mRNA expression of *TET2* (**A**), *Txnip* (**B**), and *NLRP3* (**C**) in DRG were examined. *n* = 4–5, *p < 0.05 versus DNP + Scr-siRNA group, ^#^*p* < 0.05 versus DNP + vehicle group, n.s., not significant, one-way ANOVA with Tukey’s post hoc test. **D** Representative Western blot bands for TET2 and α-tubulin in the DNP group after the intrathecal injection. **E** Statistical analysis of TET2 and α-tubulin. *n* = 6, *p < 0.05 versus DNP + Scr-siRNA group, ^##^*p* < 0.01 versus DNP + vehicle group, one-way ANOVA with Tukey’s post hoc test. **F** Representative Western blot bands for NLRP3 and α-tubulin in different groups. **G** Statistical analysis of NLRP3 and α-tubulin. *n* = 8. *p < 0.05 versus DNP + Scr-siRNA, ^#^*p* < 0.05 versus DNP + vehicle group, one-way ANOVA with Tukey’s post hoc test. **H** Intrathecal injection of TET2 significantly relieved mechanical allodynia in DNP group. n = 10–11. ***p < 0.001 versus DNP + Scr-siRNA, ###*p* < 0.001 versus DNP + Vehicle, two-way ANOVA with Bonferroni post hoc test. Experiments were repeated at least 3 times independently

In order to study whether the upregulation of NLRP3 inflammasome was mediated by TET2, *TET2*-siRNA was intrathecal injected into STZ-diabetic mice and the TXNIP–NLRP3 expression was evaluated. As depicted in Fig. 5A–C, the intrathecal injection of *TET2*-siRNA for three consecutive days began at 3 weeks after STZ injection significantly decreased the mRNA expression of *TET2* and *Txnip*, while having no effect on *NLRP3* mRNA expression in the DRG of DNP group compared to the DNP group that was administered Scr-siRNA or vehicle. In addition, by Western blot we also found the intrathecal injection of *TET2*-siRNA markedly decreased the TET2 and NLRP3 expression compared to the DNP group that was administered Scr-siRNA or vehicle (Fig. 5D–G). Using von Frey hairs, it was revealed that the intrathecal injection of *TET2*-siRNA also relieved pain sensitivity compared to the Scr-siRNA and vehicle groups (Fig. 5H).

These results demonstrated that TET2 contributed to the upregulation/activation of NLRP3 in the DNP group mice DRG mainly through upregulation of *Txnip* expression.

### High glucose exposure induced the upregulation of *Txnip* in mouse primary DRG cultures

**Fig. 6:**
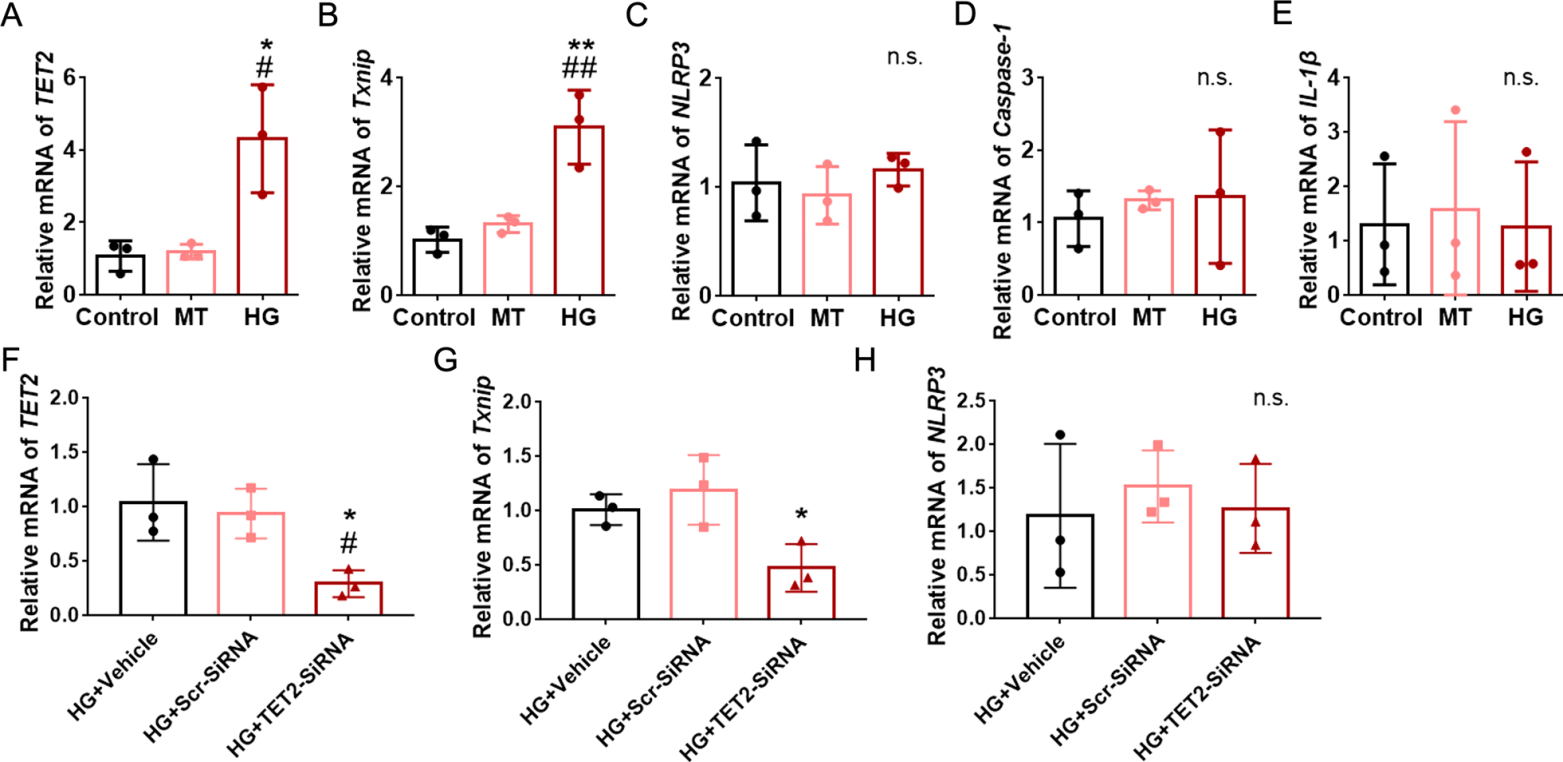
High glucose upregulated *Txnip* expression in mouse primary DRG cultures. **A**–**E** The mRNA level of *TET2* (**A**), *Txnip* (**B**), *NLRP3* (**C**)*, Caspase-1* (**D**), and *IL-1β* (**E**) in mouse primary DRG cultures under different treatments. *n* = 3, *p < 0.05, **p < 0.01 versus control; ^#^*p* < 0.05, ^##^*p* < 0.01 versus MT; n.s., not significant, one-way ANOVA with Tukey’s post hoc test. **F**–**H** *TET2*-siRNA or control siRNA was incubated with mouse primary DRG neurons cultured in high glucose (HG) condition, and the mRNA expression of *TET2* (**F**), *Txnip* (**G**), and *NLRP3* (**H**). *n* = 3, *p < 0.05 versus HG + Scr-siRNA; ^#^*p* < 0.05 versus HG + Vehicle; n.s., not significant; one-way ANOVA with Tukey’s post hoc test. Experiments were repeated 3 times independently

In order to further investigate the effect of TET2 on the upregulation of the *Txnip* in diabetic mice DRG neurons, we used HG (25 mM glucose without insulin) to culture DRG cells to mimic diabetes in vitro. Compared to the control (normal glucose; 5 mM glucose and 0.6 nM insulin) and mannitol (MT, 5 mM glucose, 0.6 nM insulin, and 20 mM MT; to exclude the effect of osmotic pressure) groups, HG significantly increased the mRNA level of *TET2* and *Txnip*, but not the mRNA level of *NLRP3*, *Caspase-1*, and *IL-1β* (Fig. 6A–E). In addition, we transfected mouse primary DRG neurons that were cultured in HG with siRNA of *TET2*. As expected, knocking down of *TET2* decreased the expression of *Txnip* markedly, but not NLRP3 (Fig. 6F–H). Moreover, we cultured DRG neurons with commonly used Neurobasal media (25 mM glucose), and set the HG concentration as 45 mM glucose in total. Similar results were observed that HG-induced the overexpression of *TET2* and subsequent upregulated *Txnip* expression in DRG neurons (Additional file 1: Fig. S2A-H). All these in vitro strongly support the in vivo results. Meanwhile, these results also indicate that the changes in the TET2–TXNIP–NLRP3 inflammasome axis we detected in vivo are likely due to the changes in neurons, but not in DRG immune cells.

### TET2 also upregulated/activated the TXNIP–NLRP3 inflammasome in the female diabetic mice

**Fig. 7:**
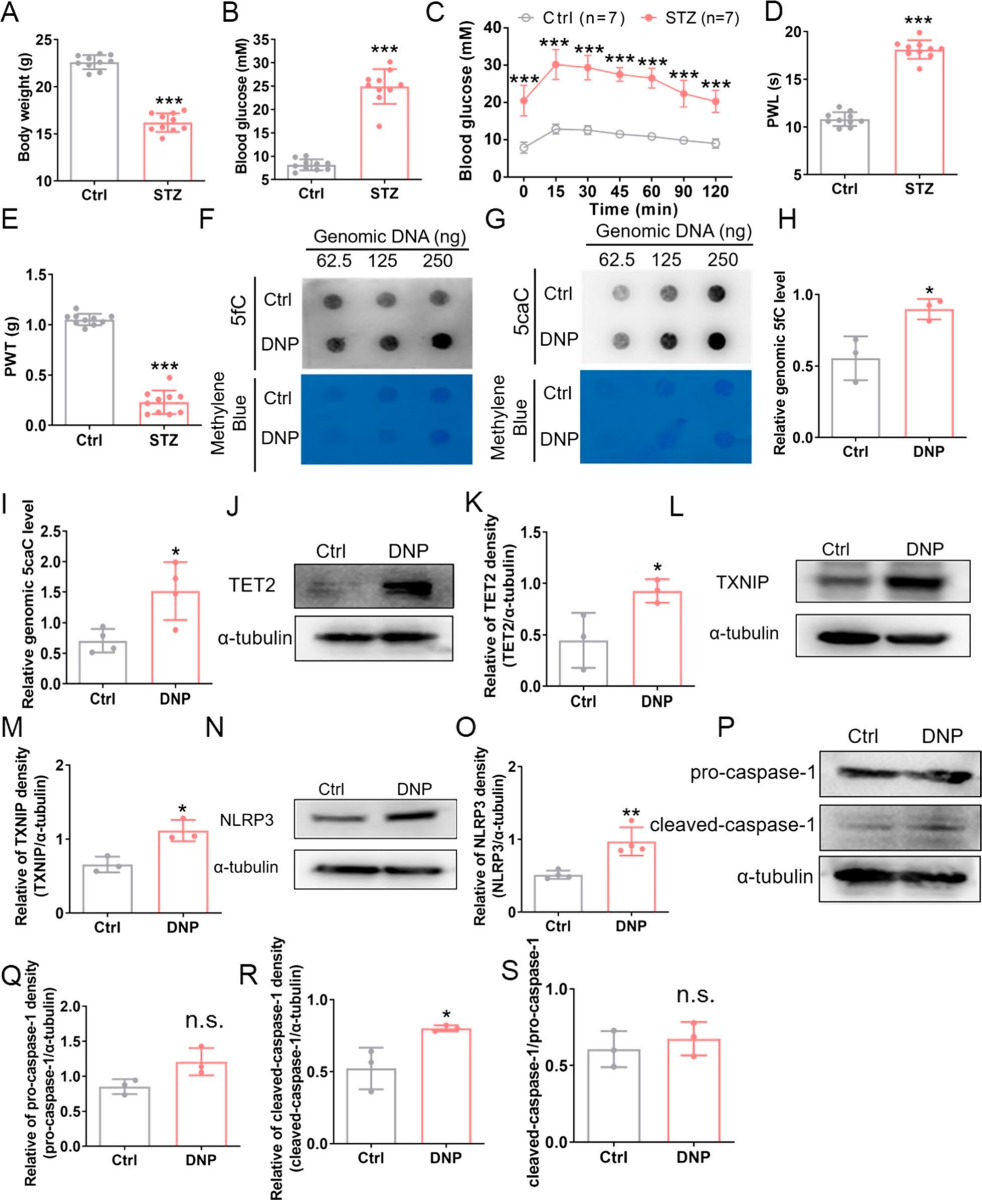
TET2 activated/upregulated TXNIP/NLRP3 in female mice. **A** Bodyweight in female mice at four weeks after STZ injection. *n* = 10, ***p < 0.001, unpaired *t*-test. **B** Fasting blood glucose in female mice at 4 weeks after STZ injection. *n* = 10, ***p < 0.001, unpaired *t*-test. **C** Glucose tolerance was measured in STZ-treated and control female mice at four weeks after STZ injection. *n* = 7, ***p < 0.001, two-way ANOVA with Bonferroni post hoc test. **D** Thermal hyposensitivity in the paws of the STZ-treated mice. *n* = 10, ***p < 0.001, unpaired *t*-test. **E** Mechanical allodynia in the paws of STZ-treated mice. *n* = 10, ***p < 0.001, unpaired *t*-test. **F**–**I** Genomic DNA 5fC and 5caC were examined in control and female STZ-diabetic mice. *p < 0.05, *n* = 3–4, unpaired *t-*test. **J**-**S** Representative Western blot bands and quantifications of the protein expression of TET2, TXNIP, NLRP3, pro-caspase-1, and cleaved-caspase-1 in female DRG tissue at 4 weeks of STZ injection, *p < 0.05, **p < 0.01, n.s., not significant; unpaired *t*-test. *n* = 3–4. Experiments were repeated at least 3 times independently

To test whether the TET2-regulated activation of TXNIP–NLRP3 inflammasome also existed in female mice, we injected STZ into female mice and examined the expression of TET2 and the components of TXNIP–NLRP3 inflammasome at four weeks after STZ injection. We first examined the body weight, blood glucose, glucose tolerance, and mechanical allodynia in the STZ-injected female mice, and found that the DNP model was successfully established in female mice at four weeks post-injection (Fig. 7A–E). We further assessed the DNA demethylation levels and found that the levels of 5fC and 5caC were increased in female mice DRG after STZ injection (Fig. 7F–I). In addition, compared with that in control DRG, the protein level of TET2 and TXNIP were markedly increased in the DNP group, and also the protein level of NLRP3 and cleaved-caspase-1 (Fig. 7J–S). These data indicate that the TET2–TXNIP–NLRP3 inflammasome axis plays a vital role in the DNP without gender difference.

## Discussion

DNP poses a great challenge in clinical treatment due to its complex and incomprehensible underlying mechanism. Research on the modulating function of environmental factors on the pain system has recently evolved, and mounting evidence suggests epigenetic modifications to be one of these modulators [35, 36]. In recent years, increasing studies have shown that aberrant DNA methylation is associated with the pain mechanism in the central nervous system [37]. Here, we showed that methylation of the whole genomic DNA was downregulated and the expression of DNA demethylase TET2 was increased in peripheral DRG of STZ-diabetic mice. The upregulation of TET2 led to the increase of mRNA level of *Txnip* and subsequent activation of the NLRP3 pathway, which contributed to the mechanical allodynia of STZ-diabetic mice.

DNA demethylation is usually mediated by ten-eleven translocation (TET) proteins, including TET1, TET2, and TET3. TET1-3 has redundant functions and are all involved in pain, but might respond to different stimulates. The studies on spared nerve injury (SNI) and spinal nerve ligation (SNL) animal models found that the levels of *TET3* mRNA in the DRG were significantly increased, while the levels of *TET1* and *TET2* exhibited no change [38, 39], indicating that TET3 having a vital role in neuropathic pain. In another study, the overexpression of TET1 in the DRG significantly alleviates SNL-induced pain hypersensitivity [40]. Moreover, the use of high levels of glucose to cultivate human mesangial cells (HMCs) or db/db diabetic mice was observed to increase the mRNA and protein levels of *TET2*, while the levels of *TET1* and *TET3* exhibited no significant difference [18]. These results taken together with our present findings indicate that under different pain conditions, the TET1-3 plays different roles in neuropathic pain, and TET2 has a higher sensitivity to stimulation with high glucose levels compared to TET1 and TET3. In this perspective, the involvement of TET2 in DNP appears plausible.

The function of TET2 is to catalyze the conversion of 5-methylcytosine (5mC) into 5-hydroxymethylcytosine (5hmC), 5-formylcytosine (5fC), and finally, 5-carboxycytosine (5caC). As revealed by immunofluorescence staining in the present study, TET2 was located mainly in the nucleus of the DRG neurons, especially in the NF200^+^ neurons. Therefore, the nuclear localization of TET2 in neurons was consistent with its function. In addition to the nuclei of DRG neurons, research reported that TET2 was also located in the nuclei of non-neuronal cells [38]. Although the authors did not perform immunostaining, they speculated that TET2 most probably colocalized with satellite and Schwann cells [38]. In our current study, although we did not investigate the localization and changes of TET2 in satellites in DRG, we cultured mouse primary DRG neurons and confirmed our in vivo results in vitro, indicating that TET2 regulates *Txnip* expression mainly in DRG neurons. Agreeing with our results, another study reported that in DRG, NLRP3 was primarily located in the neuron nuclei and not in the satellite glial cells [9]. NLRP3 is the key molecule within the inflammasome. Abundant evidence demonstrates that NLRP3 inflammasome is involved in pain conditions [41]. Upon activation, NLRP3 inflammasome produces cleaved-IL-1β from pro-IL-1β, and cleaved-IL-1β is an inflammatory cytokine capable of inducing pain [41–43]. Although the involvement of NLRP3 inflammasome in DNP has been reported in several studies [11, 44], the epigenetic mechanisms of upregulation/activation of NLRP3 inflammasome in DNP remain unclear so far.

Studies reported high levels of glucose to upregulate the expressions of TET2 and NLRP3 [18, 19]. In the GM-CSF-mediated monocyte-to-macrophage differentiation, certain inflammasome genes, such as *IL-1β*, *NLRC5,* and *AIM2,* undergo DNA demethylation, and *TET2*-siRNA is capable of impairing this demethylation and thereby impede the expression of the inflammasome-associated genes [20]. Another recent research also reveals that in cerebral ischemia/reperfusion, TET2 demethylated LncRNA TUG1, then subsequently promoted NLRP3 inflammasome and contributed to the inflammatory response [45]. These findings suggest that TET2 might be involved in the upregulation of NLRP3 inflammasome expression/activation. However, there were also research that suggested that TET2 can inhibit the expression of NLRP3 [46, 47]. Therefore, the regulation of TET2 on NLRP3 inflammasome is still uncertain. In the present study, we observed that the protein expressions of both TET2 and NLRP3 were upregulated in the STZ-diabetic mice DRG, however, the mRNA level of *NLRP3* was neither upregulated in high glucose-incubated DRG neurons in vitro, nor changed in the *TET2*-siRNA-treated DRGs in vitro and in vivo. TXNIP is an endogenous activator of the NLRP3 inflammasome, and studies had indicated that TXNIP/NLRP3 pathway contributed to the pain [48, 49]. In the present study, we found that *TET2*-siRNA could downregulate the gene expression of *Txnip* and relieve DNP. These results indicated that TET2 was involved in the upregulation of TXNIP, which then activated the NLRP3 inflammasome to contribute to DNP*.*

Studies found that the expression of NLRP3 in the sciatic nerves of DNP was increased [10, 50] and high glucose-induced the expression of NLRP3 increase in Schwann cell line RSC96 [50, 51]. These results suggested that NLRP3 in Schwann cells might also play a role in DNP. We also noticed that a report indicates that the upregulation of DNMT1 and DNMT3a mediated the increase of TXNIP in high glucose-stimulated RSC96 cells [50]. We immunostained the expression of TET2 and NLRP3 on the sciatic nerve and Schwann cells, and found that although TET2 and NLRP3 colocalized with S100 (a Schwann cell marker), there was no markedly increased expression of TET2 and NLRP3 were noticed at four weeks post-STZ injection (data not shown). Thus, whether TET2–TXNIP–NLRP3 also plays a role in the sciatic nerve and Schwann cells still needs further investigation.

Besides DNA methylation, another possible basis of the epigenetic regulation of NLRP3 expression is the non-coding RNAs [52], which may suppress or increase the gene expression by binding to the mRNA of the inflammasome. In 2012, Bauernfeind et al*.* became the first to identify miR-233–3p as the human miRNA that could regulate NLRP3 [53]. Since then, an increasing number of miRNAs are being confirmed to have a vital role in regulating the expression of NLRP3, such as miR-17–5p, miR-7, and miR-133a [54–56]. In the present study, RNA-sequencing revealed that the expression of a few non-coding RNAs was also altered in STZ-diabetic mice DRG, such as snhg7, snhg20, lpw, etc. These non-coding RNAs could also be involved in the regulation of NLRP3, although further studies are required to confirm that.

Type 2 diabetes (non-insulin dependent) is more prevalent when compared to type 1 diabetes (insulin dependent). Although the expression of TET2 in DRG in type2 diabetes had not been investigated, studies revealed that the expression of NLRP3 in DRG in type 2 diabetes was increased [11]. High glucose is a common and crucial factor in type 2 or type 1 diabetes pathologies. In our research, although we only used type 1 diabetes mouse model, we believe that the DNP in type 2 diabetes mouse would share the same TET2–TXNIP–NLRP3 axis mechanisms. We also found that gender does not affect the expression of TET2, TXNIP, and NLRP3. These results further indicated that TET2 upregulation NLRP3 through *Txnip* depended more on high glucose, but not other factors.

## Conclusions

This present study revealed that the expression/activation of NLRP3 inflammasome in the DRG neurons was dramatically increased at 4 weeks after STZ injection and contributed to mechanical pain sensitivity. Neuronal TET2 exhibited a significantly increased expression and was involved in the mechanical pain occurring in STZ-diabetic through regulating *Txnip* gene expression directly and then influencing expression/activation of NLRP3 inflammasome. In addition, we proposed that both silencing *TET2* or directly inhibiting NLRP3 inflammasome activation effectively decrease DNP, and may serve as future therapeutic strategies in the clinic.

## Supplementary Information

**Additional file 1: Fig. S1. **Reduction of epidermal fibers in footpads of diabetic mice at 8 weeks post-STZ injection. **A** Representative images of the intraepidermal nerve fiber profiles in nondiabetic control and diabetic mice. The positive staining of nerve fibers appears tan-brown and the cells are stained light blue. **B** Quantification of the intraepidermal nerve fiber profiles. *n* = 8, ** *p *< 0.01 versus Control, Two-tailed Student’s *t* test followed by Tukey's multiple comparisons test. Scale bar: 20 μm. Experiments were repeated at least 3 times independently. **Fig. S2. **High glucose upregulation of *Txnip* expression in mouse primary DRG cultures. **A**–**E** The mRNA level of *TET2* (**A**), *Txnip* (**B**), *NLRP3 *(**C**)*, Caspase-1* (**D**), and *IL-1β *(**E**) in mouse primary DRG cultures under different treatments. n = 3, *p < 0.05 versus control; ^#^*p *< 0.05, ^##^*p *< 0.01 versus MT; n.s., not significant, one-way ANOVA with Tukey’s post hoc test. **F**–**H** *TET2*-siRNA or control siRNA was incubated with mouse primary DRG neurons cultured in high glucose (HG) condition, and the mRNA expression of *TET2 *(**F**), *Txnip* (**G**), and *NLRP3* (**H**). *n* = 4, *p < 0.05 versus HG + Scr-siRNA; ^#^*p *< 0.05 versus HG + Vehicle; n.s., not significant, one-way ANOVA with Tukey’s post hoc test. Experiments were repeated at least 3 times independently.

## Data Availability

All data generated or analyzed during this study are included in the published article (and its additional files) and are available from the corresponding author upon reasonable request. The raw RNA-sequencing (RNA-seq) generated during and/or analyzed during the current study is available from the corresponding author upon reasonable request.

## Abbreviations

AIM2: Absence in melanoma 2
ALRs: Absence in melanoma 2-like receptors
ANOVA: Analysis of variance
DNMT: DNA methyltransferase
DNP: Diabetic neuropathic pain
DRG: Dorsal root ganglion
HG: High glucose
HMCs: Human mesangial cells
GTT: Glucose tolerance test
HUVECs: Human umbilical vein endothelial cells
INFD: Intraepidermal nerve fiber density
*i.p*.: Intraperitoneal
*i.t*.: Intrathecal
KEGG: Kyoto Encyclopedia of Genes and Genomes
MT: Mannitol
NLRs: Nucleotide-binding domain, leucine-rich repeat-containing proteins
NLRP3: Nucleotide oligomerization domain-like receptor family pyrin domain containing 3
PWL: Paw withdrawal latency
PWT: Paw withdrawal threshold
SNI: Spared nerve injury
SNL: Spinal nerve ligation
STZ: Streptozocin
TXNIP: Thioredoxin-interacting protein
TETs: Ten-eleven translocation methylcytosine dioxygenases
5caC: 5-Carboxycytosine
5fC: 5-Formylcytosine
5hmC: 5-Hydroxymethylcytosine
5mC: 5-Methylcytosine
