# Supplementary material for: The upregulation of NLRP3 inflammasome in dorsal root ganglion by ten-eleven translocation methylcytosine dioxygenase 2 (TET2) contributed to diabetic neuropathic pain in mice
Source: J Neuroinflammation. 2022 Dec 16;19:302. doi: 10.1186/s12974-022-02669-7 (PMC9756585; doi:10.1186/s12974-022-02669-7)
Supplement: Supplementary file 1 — Additional file 1: Fig. S1. Reduction of epidermal fibers in footpads of diabetic mice at 8 weeks post-STZ injection. A Representative images of the intraepidermal nerve fiber profiles in nondiabetic control and diabetic mice. The positive staining of nerve fibers appears tan-brown and the cells are stained light blue. B Quantification of the intraepidermal nerve fiber profiles. n = 8, ** p < 0.01 versus Control, Two-tailed Student’s t test followed by Tukey's multiple comparisons test. Scale bar: 20 μm. Experiments were repeated at least 3 times independently. Fig. S2. High glucose upregulation of Txnip expression in mouse primary DRG cultures. A–E The mRNA level of TET2 (A), Txnip (B), NLRP3 (C), Caspase-1 (D), and IL-1β (E) in mouse primary DRG cultures under different treatments. n = 3, *p < 0.05 versus control; #p < 0.05, ##p < 0.01 versus MT; n.s., not significant, one-way ANOVA with Tukey’s post hoc test. F–H TET2-siRNA or control siRNA was incubated with mouse primary DRG neurons cultured in high glucose (HG) condition, and the mRNA expression of TET2 (F), Txnip (G), and NLRP3 (H). n = 4, *p < 0.05 versus HG + Scr-siRNA; #p < 0.05 versus HG + Vehicle; n.s., not significant, one-way ANOVA with Tukey’s post hoc test. Experiments were repeated at least 3 times independently. [file 12974_2022_2669_MOESM1_ESM.docx]

**Additional file 1**

**Additional Figures and Legends**

**
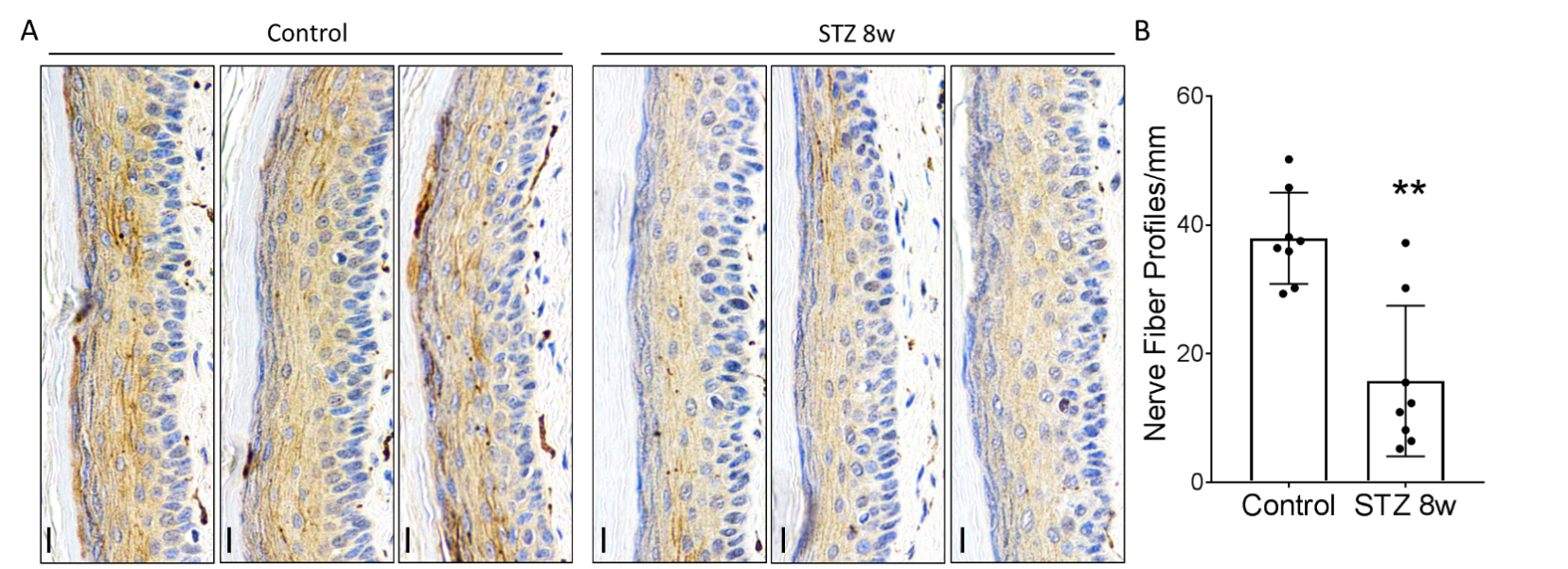
**

**Fig. S1. Reduction of epidermal fibers in footpads of diabetic mice at 8 weeks post-STZ injection.** (A) Representative images of the intraepidermal nerve fiber profiles in nondiabetic control and diabetic mice. The positive staining of nerve fibers appears tan-brown and the cells are stained light blue. (B) Quantification of the intraepidermal nerve fiber profiles. n=8, ** *p* < 0.01 versus Control, Two-tailed student’s *t* test followed by Tukey's multiple comparisons test. Scale bar: 20 μm. Experiments were repeated at least 3 times independently.


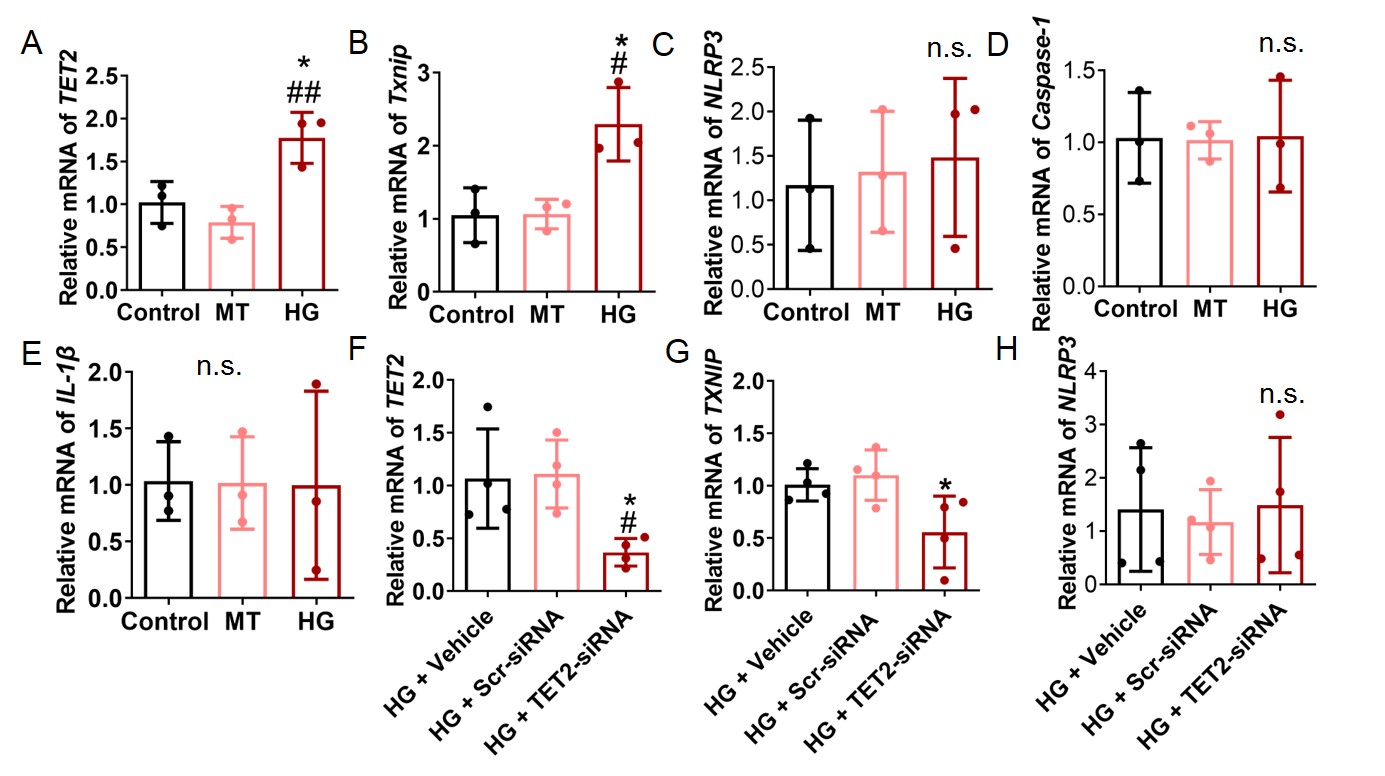


**Fig. S2. High glucose upregulation of *Txnip* expression in mouse primary DRG cultures.** (A-E) The mRNA level of *TET2* (A), *Txnip* (B), *NLRP3* (C*), Caspase-1* (D), and *IL-1β* (E) in mouse primary DRG cultures under different treatments. n=3, **p* < 0.05 versus control; #*p* < 0.05, ##*p* < 0.01 versus MT; n.s., not significant, one-way ANOVA with Tukey’s post hoc test. (F-H) *TET2*-siRNA or control siRNA was incubated with mouse primary DRG neurons cultured in high glucose (HG) condition, and the mRNA expression of *TET2* (F), *Txnip* (G), and *NLRP3* (H). n=4, **p* < 0.05 versus HG + Scr-siRNA; #*p* < 0.05 versus HG + Vehicle; n.s., not significant, one-way ANOVA with Tukey’s post hoc test. Experiments were repeated at least 3 times independently.
